# A deep neural network framework to derive interpretable decision rules for accurate traumatic brain injury identification of infants

**DOI:** 10.1186/s12911-023-02155-x

**Published:** 2023-04-06

**Authors:** Baiming Zou, Xinlei Mi, Elizabeth Stone, Fei Zou

**Affiliations:** 1 Department of Biostatistics, University of North Carolina at Chapel Hill, Chapel Hill, NC 27599 USA; 2 School of Nursing, University of North Carolina at Chapel Hill, Chapel Hill, NC 27599 USA; 3 Department of Preventive Medicine - Biostatistics Quantitative Data Sciences Core (QDSC), Northwestern University, Chicago, IL 60611 USA; 4 Department of Genetics, University of North Carolina at Chapel Hill, Chapel Hill, NC 27599 USA

**Keywords:** Deep neural network, Diagnosis test, Feature importance, Head trauma, Testable machine learning, Permutation

## Abstract

**Objective:**

We aimed to develop a robust framework to model the complex association between clinical features and traumatic brain injury (TBI) risk in children under age two, and identify significant features to derive clinical decision rules for triage decisions.

**Methods:**

In this retrospective study, four frequently used machine learning models, i.e., support vector machine (SVM), random forest (RF), deep neural network (DNN), and XGBoost (XGB), were compared to identify significant clinical features from 24 input features associated with the TBI risk in children under age two under the permutation feature importance test (PermFIT) framework by using the publicly available data set from the Pediatric Emergency Care Applied Research Network (PECARN) study. The prediction accuracy was determined by comparing the predicted TBI status with the computed tomography (CT) scan results since CT scan is the gold standard for diagnosing TBI.

**Results:**

At a significance level of $p = 0.05$, DNN, RF, XGB, and SVM identified 9, 1, 2,  and 4 significant features, respectively. In a comparison of accuracy (Accuracy), the area under the curve (AUC), and the precision-recall area under the curve (PR-AUC), the permutation feature importance test for DNN model was the most powerful framework for identifying significant features and outperformed other methods, i.e., RF, XGB, and SVM, with Accuracy, AUC, and PR-AUC as 0.915, 0.794, and 0.974, respectively.

**Conclusion:**

These results indicate that the PermFIT-DNN framework robustly identifies significant clinical features associated with TBI status and improves prediction performance. The findings could be used to inform the development of clinical decision tools designed to inform triage decisions.

## Introduction

Head trauma often occurs in very young children, accounting for 1.62% of emergency department (ED) visits annually in the United States [1–3]. Children under age 2 represent approximately 25% of ED visits for head trauma in the United States [4]. Compared to older children, these age group children are more susceptible to skull fracture and intracranial injury (traumatic brain injury [TBI]) due to several anatomical and physiological differences [5, 6]. When undetected, these injuries may lead to complications including cognitive impairment or even death and disabilities in the future [7]. The triage nurse is typically the first healthcare professional to assess children who present to an ED for evaluation and is tasked with an acuity decision that guides the initial prioritization of patient care. A major concern of the triage nurse is: in head-injured children under age 2 who appear to have age-appropriate or near age-appropriate mental status on exam, which ones are at a high risk of TBI so that they should be assigned a high acuity level to expedite an evaluation by a medical provider who will determine the need for computed tomography (CT) imaging to verify TBI status [8]? Effective clinical decision rules based on clinical features (i.e., significant clinical features that can be used to appropriately determine TBI risk) to aid the ED triage nurse are therefore critical, however, challenging to determine. A particular obstacle is that among many clinical features which are significant features that can be used to distinguish TBI status in children under age two, who are not only often the most difficult to assess but are also at the highest risk of TBI? Correctly identifying significant features is useful in the ED setting since it can help nurses to focus on examining these features and make appropriate triage decisions promptly. Another difficulty is that several features including age, location of the injury, mechanism of injury, etc., jointly impact the TBI status for these children in complex fashions [6, 8–10]. Furthermore, children in this age group are particularly difficult to assess since they have limited verbal ability to explain what happened and usually demonstrate developmental anxiety [8]. Though great efforts have been made to assess clinical features that can reliably predict the need for CT imaging in pediatric head trauma to verify TBI status, the conclusions are inconsistent[11, 12] or even conflicting, e.g., a poor correlation between the clinical symptoms of significant TBI and findings on CT was identified [13]. Many children under age 2 who have sustained a TBI are clinically asymptomatic [10].

Though a few clinical decision rules (i.e., CATCH [14] and CHALICE [15]) have been derived to aid the medical provider in their neuroimaging decision for children, these decision rules were determined by using a simple univariate $\chi ^{2}$ test to identify those statistically significant clinical features one by one. Like existing statistical methods for detecting the significance of each clinical feature associated with TBI, they all adopted logistic regression with (generalized) linear additive assumptions [16–20]. However, these restrictive assumptions often do not hold and are difficult to verify in practice [16, 21]. Furthermore, the univariate method did not adjust for confounding factors that could lead to biased parameter estimates and overfitting, i.e., the prediction accuracy for future children using the decision rules could be low. More robust machine learning methods, e.g., the classification and regression tree (i.e., PECARN rule [6]) and the optimal classification tree, [22] were adopted to derive the clinical decision rules for TBI identification in children. Though these decision rules relax those very restrictive assumptions made in the parametric method, all these rules were derived without conducting any statistical test, i.e., the “importance” of each clinical feature was empirically determined. Therefore, the role of each clinical feature included in these rules is not necessarily significant and not statistically interpretable either. Identifying significant clinical features associated with TBI for ED acuity decision-making is critical yet challenging because many clinical features can interactively impact TBI status in very complex fashions. To address this challenge and motivated by deep neural network (DNN) models for accurately approximating complex functions universally, [23, 24] we adopt a scoring algorithm to filter out unstable DNN models due to the random parameter initialization used in the conventional DNN method [25, 26] to deal with the complexity of TBI data and improve the prediction performance. However, like all other machine learning models, DNN-based methods suffer from the interpretation transparency of each clinical feature’s role in outcome prediction due to the use of abstract algorithm, i.e., they are black-box machines without the capability to assess the exact significance of each predictor in the model. The black-box nature of machine learning models may introduce confusion [27]. To overcome this disadvantage, we further adopt a permutation-based feature importance test (PermFIT) procedure for the DNN model with a valid and robust statistical test to correctly identify significant features for predicting TBI status reliably [28]. PermFIT has shown to be a powerful tool for identifying important features for different data types, and applicable to various machine learning models [28, 29].

The rest of the paper is organized as follows: we describe the details of a scoring algorithm to construct a stable DNN ensemble for robustly modeling the complex association relationship between clinical features and TBI status along with a universal feature importance test procedure, i.e., PermFIT, for deciphering the role of each clinical feature on developing TBI risk in the Methods section. We then apply the derived PermFIT-DNN framework to a cohort of children under age 2 with suspected minor head trauma to derive statistically significant clinical features associated with TBI risk in the Results section. A brief discussion concludes the paper.

## Methods

**TBI Prediction via Machine Learning Models:**  Let $X = (X_1, ..., X_p)$ be a *p*-dimensional clinical features (e.g., age, injury mechanism, etc), and *Z* be the binary TBI status (e.g., $Z=1$ and 0 for positive and negative, respectively) with $\pi (X) = \text {E}(Z|X) = \text {Pr}(Z=1|X)$ being the conditional probability of positive TBI given the observed clinical features *X*. To predict TBI status, we need to obtain the estimate of $\pi (X=x)$, i.e., $\hat{\pi }(x)$. Traditionally, a logistic regression model is used. This modeling strategy, however, needs to make the (generalized) linear additive assumptions for the clinical features, meaning these features impact the probability of positive TBI status in a generalized linear additive fashion. That is under a logistic regression framework, the logit of the probability to be in positive TBI status is assumed to be a function of the linear combination of the clinical features. However, the linear additive assumption can barely hold and is difficult to verify in practice. To relax this restrictive assumption, machine learning methods can be adopted. Indeed, tree-based machine learning methods have been used to derive clinical decision rules for TBI status identification [6, 22]. Here, we compare several commonly used machine learning models, i.e., deep neural network (DNN) [30, 31], random forest (RF) [32, 33], XGBoost (XGB) [34] and support vector machine (SVM) [35, 36], for their performance in identifying the statistically significant features associated with TBI status. However, unlike the conventional DNN method, we introduce a scoring algorithm to address unstable prediction issue of conventional DNN method as we describe below.

**Scoring Algorithm for Constructing Stable DNN Ensemble:**  Due to the random parameter initialization used in the conventional DNN method, it could result in unstable predictions. Here, we adopt the stable DNN procedure by using a scoring algorithm [25] to filter out those unstable bootstrapped DNN models. To avoid the redundancy, we refer readers to the paper by LeCun et al. for the general review about conventional DNN method [31]. The stable DNN method can remarkably improve prediction precision [26]. Specifically, we adopt the following score-based algorithm to filter out poorly performing DNN models as described following.

That is, instead of assuming $\pi (\mathbf {X=x})$ being a linear additive function of **x**, we relax this assumption by letting $\pi (\mathbf {X=x})$ being an unspecified smooth function of **x**, and use an *L*-hidden-layer feedforward DNN model to approximate it since DNN is an ideal tool for universally approximating very complex functions [31]. Let the output function be $g_\text {out}(\tau ) = 1/(1+e^{-\tau })$, and the inner activation functions be $\varvec{g}_l ~ (l=1, \cdots , L)$, e.g., a rectified linear activation unit function (ReLU), [37] leading to the final convoluted output function as $\pi = g_\text {out} \circ \varvec{g}_L \circ \cdots \circ \varvec{g}_1$, with $\varvec{\theta }= \{\varvec{b}^{(i)}, \varvec{W}^{(i)}\}_{i=1}^{L+1}$ being a parameter vector and estimated by minimizing the associated risk function. The risk function associated with the DNN model is optimized via a mini-batch stochastic gradient descent algorithm [38, 39], along with the adaptive learning rate adjustment [40]. However, DNN could be unstable in finite sample settings. Here, we adopt two procedures, i.e., bootstrapping and filtering, to address this issue. First, the bootstrap aggregating [41] is adopted to increase the stability and accuracy of a single DNN [42]. To further boost DNN performance, we adopt a filtering algorithm to remove poorly performing bagged DNNs based on the observation that ensembling only a subset of bagged DNNs that are well fit to the data could lead to a better performing ensemble model [25, 43]. Specifically, the filtering algorithm first calculates the performance scores $\{\lambda _k\}_{k=1}^{K}$ associated with the *K* sets of bootstrapped samples as follows:

$$\begin{aligned} \lambda _k = \sum \limits _{i\in \mathcal {D}_{{O_k}}}\frac{1}{n_{{O_k}}} \left\{ y_i\log \left( \frac{\widehat{y}_{i{k}}}{\overline{y}_{O_k}}\right) + (1-y_i)\log \left( \frac{1-\widehat{y}_{i{k}}}{1-\overline{y}_{O_k}}\right) \right\} \end{aligned}$$

where $\mathcal {D}_{{O_k}}$ is the $k^{th}$ set of out-of-bag bootstrap samples with $n_{{O_k}}$ being the associated sample size, $\widehat{\pi }_{k}$ being the estimated function of $\pi$, $\widehat{y}_{ik} = \widehat{\pi }_k(X_i)$ ($i\in D_{O_k}$), and $\overline{y}_{O_k} = \sum _{i\in \mathcal {D}_{O_k}}\hat{y}_i/n_{O_k}$. Based on the performance scores, the filtering algorithm then selects an optimal subset of bagged DNNs to construct the final ensembled DNN model, where the optimal number of DNNs for the final ensemble can be determined by minimizing the training loss [25, 26]. With the scoring algorithm, only those top performing bagged DNN models will be kept in the final ensemble such that stable and accurate predictions will be obtained. Also, with the stable DNN procedure, there is no need to involve the cumbersome parameter tuning process as required in the conventional DNN method.

Though the machine learning methods relax the restrictive assumption made in the traditional parametric method and improve the prediction accuracy, they lack transparency regarding the role of each clinical feature on outcome prediction accuracy. To determine the significant features based on a valid statistical inference for machine learning models, we adopt the permutation-based feature importance test procedure as briefly described below [28].

**Significant Clinical Features Identification for TBI:** Though the permutation-based feature importance methods have been proposed for random forests and DNNs, [29, 44] they do not conduct any statistical inference on the feature importance. Instead, we adopt the following general permutation feature importance test procedure for machine learning models [28].

We define the feature importance score $\Delta _j$ of $X_j$ (i.e., the $j^{\text {th}}$ feature in *X* $(j=1,...,p)$) as the expected squared difference between $\pi (X)$ and $\pi \left( X^{(j)}\right)$, where $X^{(j)} = (X_1, ..., X_{j-1}, X_{j'}, X_{j+1}, ..., X_p)$, or *X* but with its $j^{th}$ feature replaced by $X_{j'}$, a random permutation of the elements of $X_{j}$. The importance score $\Delta _j$ can be re-expressed as $\Delta _j = \textrm{E}_{X, X_{j'}}[\pi (X)-\pi \left( X^{(j)}\right) ]^2$, which is zero only when $\pi (X)\equiv \pi (X^{(j)})$, implying no contribution of $X^{(j)}$ on $\pi (X)$ conditional on the other covariates. The stronger the impact of $X^{(j)}$ on $\pi (X)$, the larger $\Delta _j$ is expected to be. Furthermore, $\Delta _j$ can be estimated empirically. Let $X_j' = (X_{s_1,j}, ..., X_{s_n,j})$ be a random sample of the elements in $X_j$ without replacement, and the empirical permutation importance score be $\Delta _j^{(P)} = \frac{1}{n}\sum _{i=1}^n \Delta _{ij}^{(P)}$ where $\Delta _{ij}^{(P)} = Z_{i}\log \left( \frac{\widehat{\pi }(X_{i.})}{\widehat{\pi }(X_{i.}^{(j)})}\right) + (1-Z_{i})\log \left( \frac{1-\widehat{\pi }(X_{i.})}{1-\widehat{\pi }(X_{i.}^{(j)})}\right)$ with $X_{i\cdot } = (X_{i1}, ..., X_{ip})$ and $X_{i\cdot }^{(j)} = (X_{i1}, \cdots , X_{i,j-1}, X_{s_i,j}, X_{i,j+1},\cdots , X_{ip})$. Note that $\textrm{E}[\Delta _j^{(P)}] = \textrm{E}[\Delta _{ij}^{(P)}] = \frac{n-1}{n} \Delta _j$. $\pi (\cdot )$ estimate, i.e. $\widehat{\pi }(\cdot )$, can be obtained using four machine learning models we consider, i.e., DNN, RF, XGB, and SVM, or the parametric logistic regression method. Particularly, the DNN method we use is the stable DNN [25, 26] as we described above. $\Delta _j^{P}$ can then be estimated as

$$\begin{aligned} \widehat{\Delta }_j^{(P)} = \frac{1}{n}\sum \limits _{i=1}^n \left[ Z_{i}\log \left( \frac{\widehat{\pi }(X_{i.})}{\widehat{\pi }(X_{i.}^{(j)})}\right) + (1-Z_{i})\log \left( \frac{1-\widehat{\pi }(X_{i.})}{1-\widehat{\pi }(X_{i.}^{(j)})}\right) \right] \end{aligned}$$

To avoid potential overfitting of the approximator $\widehat{\pi }(\cdot )$ under the finite sample size setting, we employ a cross-fitting strategy to separate the input data into training and validation sets, with the training set used for generating $\widehat{\pi }(\cdot )$ and the testing set for estimating $\widehat{\Delta }_j^{(P)}$. Let $\widehat{\pi }_T(\cdot )$ be the estimate of $\pi (\cdot )$ from the training set, and $\mathcal {D}_V = \{Z_i, X_{i\cdot }\}_{i=1}^{n_V}$ be the validation set, we obtain the feature importance score estimate $\widehat{\Delta }_j^{(P)}$ as:

$$\begin{aligned} \widehat{\Delta }_j^{(P)} = \frac{1}{n_V}\sum \limits _{i=1}^{n_V} \left[ Z_{i}\log \left( \frac{\widehat{\pi }_{T}(X_{i.})}{\widehat{\pi }_{T}(X_{i.}^{(j)})}\right) + (1-Z_{i})\log \left( \frac{1-\widehat{\pi }_{T}(X_{i.})}{1-\widehat{\pi }_{T}(X_{i.}^{(j)})}\right) \right] \end{aligned}$$

and the variance estimate of $\widehat{\Delta }_j^{(P)}$ as:

$$\begin{aligned} \widehat{\textrm{Var}}[\widehat{\Delta }_j^{(P)}] = \frac{1}{n_V}\sum \limits _{i=1}^{n_V} \left[ Z_{i}\log \left( \frac{\widehat{\pi }_{T}(X_{i.})}{\widehat{\pi }_{T}(X_{i.}^{(j)})}\right) + (1-Z_{i})\log \left( \frac{1-\widehat{\pi }_{T}(X_{i.})}{1-\widehat{\pi }_{T}(X_{i.}^{(j)})}\right) - \widehat{\Delta }_j^{(P)}\right] ^2 \end{aligned}$$

Based on it, we construct the test statistic for importance hypothesis test of feature $X_{j}$ as:

1 $$\begin{aligned} \delta =\frac{\widehat{\Delta }_j^{(P)}}{\sqrt{\widehat{\textrm{Var}}[\widehat{\Delta }_j^{(P)}]}} \end{aligned}$$

The PermFIT-DNN procedures are summarized in Algorithm 1, and the PermFIT R package is available at https://github.com/SkadiEye/deepTL. With the PermFIT procedure, we can determine each feature’s effect on the TBI status (i.e., with the corresponding *p*-value) under the complex functional relationship using different machine learning models. Based on the evaluated *p*-values, we then can determine which are the significant clinical features and which are not, thus ED nurses can focus on those significant features to make triage decisions for children under age two with suspected minor head trauma, which is not available if the existing feature importance tools are used.

**Figure:**
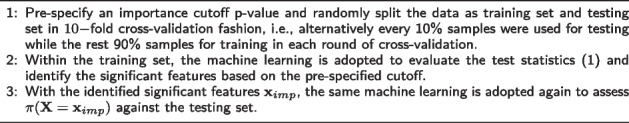
**Algorithm 1** Significant Feature Identification via Machine Learning Method

**Study Design and Participants:**   To derive the clinical decision rule for very young age children, we apply the PermFIT framework for four machine learning models and one parametric logistic regression method, to a subset of the publicly available data from the Pediatric Emergency Care Advanced Research Network (PeCARN) Head Injury Study, a federally funded cohort study of children under 18 years of age who had sustained head trauma within prior 24 hours and presented to an ED for evaluation of suspected minor head trauma [6]. The PeCARN study excluded children who had “trivial” injury mechanisms (ground-level falls or walking or running into stationary objects) with no signs of head trauma other than abrasions or lacerations. In their primary study, the researchers also excluded children from who had penetrating trauma, pre-existing neurological disorders, known brain tumors or previous neuroimaging for the injury. The definition of “suspected minor head trauma” was based on Glasgow Coma Scale scores of 14 or 15 which indicate an age-appropriate or near-age-appropriate mental status on exam. Total 42, 412 participants were enrolled on a consecutive basis from 2004 to 2006 from 25 emergency departments within a U.S. pediatric research network. Among them, 10, 718 were very young children of age under 2 years. In this study, we include the 1, 429 children of age under 2 years who had a completed CT scan (i.e., TBI status is known) without missing values for all 24 clinical features collected in the primary study (i.e., samples with missing values and without CT scan conducted are excluded from the analysis) [6]. TBI was defined by any of the following findings on CT scan: intracranial hemorrhage or contusion; cerebral edema; traumatic infarction, diffuse axonal injury; shearing injury; sigmoid sinus thrombosis; midline shift of intracranial contents or signs of brain herniation; diastasis of the skull, pneumocephalus, or skull fracture depressed by at least the width of the table of the skull [6]. Among the 1, 429 children in our sample, 122 kids were diagnosed as TBI positive by CT scan. We adopted the PermFIT framework as described above to identify the significant features to be used to predict TBI status reliably from the 24 available clinical features. They include: the children’s age in months, injury mechanism, severity of injury mechanism (i.e., low, moderate, high), history of loss of consciousness, presence of any post-traumatic seizure, duration of any post-traumatic seizure, whether they are acting normally according to their caregiver, presence of vomiting after head injury, number of vomiting episodes, altered mental status according to the ED provider, anterior fontanelle bulging, raised scalp hematoma or swelling, hematoma or swelling location, size of the largest hematoma or swelling (i.e., small (< 1cm, barely palpable), medium (1-3 cm), large (> 3cm), and not applicable), evidence of trauma (bruise, laceration or hematoma) above the clavicles, trauma above the clavicles region: face, trauma above the clavicles region: neck, trauma above the clavicles region: scalp-frontal, trauma above the clavicles region: scalp-occipital, trauma above the clavicles region: scalp-parietal, trauma above the clavicles region: scalp-temporal, gender, ethnicity, and race. Major clinical feature distribution of the 24 total clinical features is summarized in Table 1.

**Table 1:** Baseline Characteristics for Children under Age 2 who Received a CT Scan

Categorical Feature		No. (%)
Gender	Boy	765 (53.53%)
	Girl	664 (46.47%)
Post-traumatic seizure	Yes	43 (3.01%)
	No	1386 (96.99%)
Acting normally according to caregiver	Yes	962 (67.32%)
	No	467 (32.68%)
Altered mental status according to healthcare provider	Yes	416 (29.11%)
	No	1013 (70.89%)
Injury mechanism	Motor vehicle collision	31 (2.17%)
	Pedestrian struck by moving vehicle	12 (0.83%)
	Bike rider struck by automobile	1 (0.07%)
	Bike collision or fall from bike	2 (0.14%)
	Other wheeled transport crash	11 (0.77%)
	Fall from standing/walking/running	97 (6.79%)
	Walked or ran into stationary object	42 (2.94%)
	Fall from an elevation	858 (60.04%)
	Fall down stairs	188 (13.16%)
	Sports	0 (0.00%)
	Assault	15 (1.05%)
	Object struck head - accidental	72 (5.04%)
	Other	100 (7.00%)
Duration of post-traumatic seizure	$<1$ min	20 (1.40%)
	$1-<5$ min	20 (1.40%)
	$5-15$ min	3 (0.21%)
	$>15$ min	0 (0.00%)
	Not applicable	1386 (96.99%)
Hematoma or swelling location	Frontal	350 (24.49%)
	Occipital	94 (6.58%)
	Parietal/Temporal	266 (18.62%)
	Not applicable	719 (50.31%)
Size of hematoma or swelling	Small	120 (8.40%)
	Medium	391 (27.36%)
	Large	199 (13.93%)
	Not applicable	719 (50.31%)
Trauma above clavicles: scalp-frontal	Yes	341 (23.86%)
	No	488 (34.15%)
	Not applicable	600 (41.99%)
Trauma above clavicles: scalp-parietal	Yes	157 (10.99%)
	No	672 (47.02%)
	Not applicable	600 (41.99%)
**Continuous Feature**		**Mean (SD)**
Age (in month)		9.30 (6.75)

## Results

We adopt a 10-fold cross-validation by alternatively using one fold for testing and the other 9 folds for training, i.e., alternatively every 10% samples were used for testing while the rest 90% samples for training in each round of cross-validation. Under the permutation feature importance test framework, we use DNN, RF, XGB, and SVM method (referred as PermFIT-DNN, PermFIT-RF, PermFIT-XGB, and PermFIT-SVM, respectively) to identify the significant clinical features associated with TBI status at the significance level 0.05. For stable DNN method, we used four hidden layers (no dropout layer) with (50, 40, 30, 20) hidden nodes from the first to the last hidden layer, respectively. The risk function is optimized via a mini-batch stochastic gradient descent algorithm [38, 39], along with the adaptive learning rate adjustment [40]. For the random forest method, we implemented by using R package “randomForest” with 1000 trees and other tuning parameters used were based on a 5-fold cross-validation (i.e., the minimum size terminal nodes 3 and 4 variables randomly sampled as candidates at each split were determined). We adopted xgboost R package with turning parameters determined via a cross-validation (i.e., the max number of boosting iterations, 5, was determined). The tuning parameters used in SVM method were determined by using 5-fold cross-validation. Furthermore, the data used for hyper-parameter tuning and that used for performance evaluation are different. In particular, the data used for evaluating the performance of the model are never leaked/seen when training the model in each round of the cross-validation. Significant clinical features identified by each method and the corresponding *p*-values are presented in Table 2.

**Table 2:** Identified Significant Clinical Features

Method	Significant Clinical Features	*P*-value
PermFIT-DNN	Age	$<0.001$
	acting normally according to parent	0.035
	Altered mental status	0.015
	Injury mechanism	0.028
	Duration of post-traumatic seizure	0.002
	Hematoma or swelling location	$<0.001$
	Size of hematoma or swelling	0.001
	Trauma above clavicles: scalp-frontal	0.048
	Trauma above clavicles: scalp-parietal	0.042
PermFIT-RF	Age	0.001
PermFIT-SVM	Age	0.043
	Altered mental status	0.019
	Hematoma or swelling location	$<0.001$
	Size of hematoma or swelling	0.003
PermFIT-XGBoost	Age	$<0.001$
	Size of hematoma or swelling	0.022

Results of Table 2 clearly indicate that the PermFIT-DNN is the most powerful method among all machine learning models for detecting the significant clinical features associated with the TBI status. While the PermFIT-DNN method claims 9 clinical features as significant features, the PermFIT-SVM, PermFIT-RF, and PermFIT-XGB methods claim 4, 1, and 2 features, respectively, as the significant features. Among the identified significant features by all machine learning models, we notice that age is the only clinical feature that is commonly claimed by all methods as the significant feature.

With the selected significant features by each method, we evaluate the performance for predicting TBI status and draw a comparison with the corresponding machine learning model (i.e., DNN, RF, SVM, and XGB) using all 24 clinical features for predicting TBI status via 10-fold cross-validation. In evaluating the performance, we adopt three metrics including accuracy (Accuracy), the area under the receiver operating characteristic curve (AUC), and the precision-recall area under the curve (PR-AUC). For the accuracy evaluation, we use the cutoff of 0.5. That is the TBI status (i.e. $z_{i}$) for a patient *i* with clinical features $X=x$ is predicted as the following:

$$\begin{aligned} \hat{z}_{i} = \left\{ \begin{array}{ll} 1 &{} ~~~~~\text {if }~\hat{\pi }_{i}(x) > 0.5\\ 0 &{} ~~~~~\text {if }~\hat{\pi }_{i}(x) \le 0.5\\ \end{array} \right. \end{aligned}$$

where $\hat{\pi }_{i}(x)$ is the predicted probability for sample *i* being in positive TBI status based on the corresponding machine learning method employed conditioning on the clinic features included in the model. Performance comparisons are presented in Table 3.

**Fig. 1:**
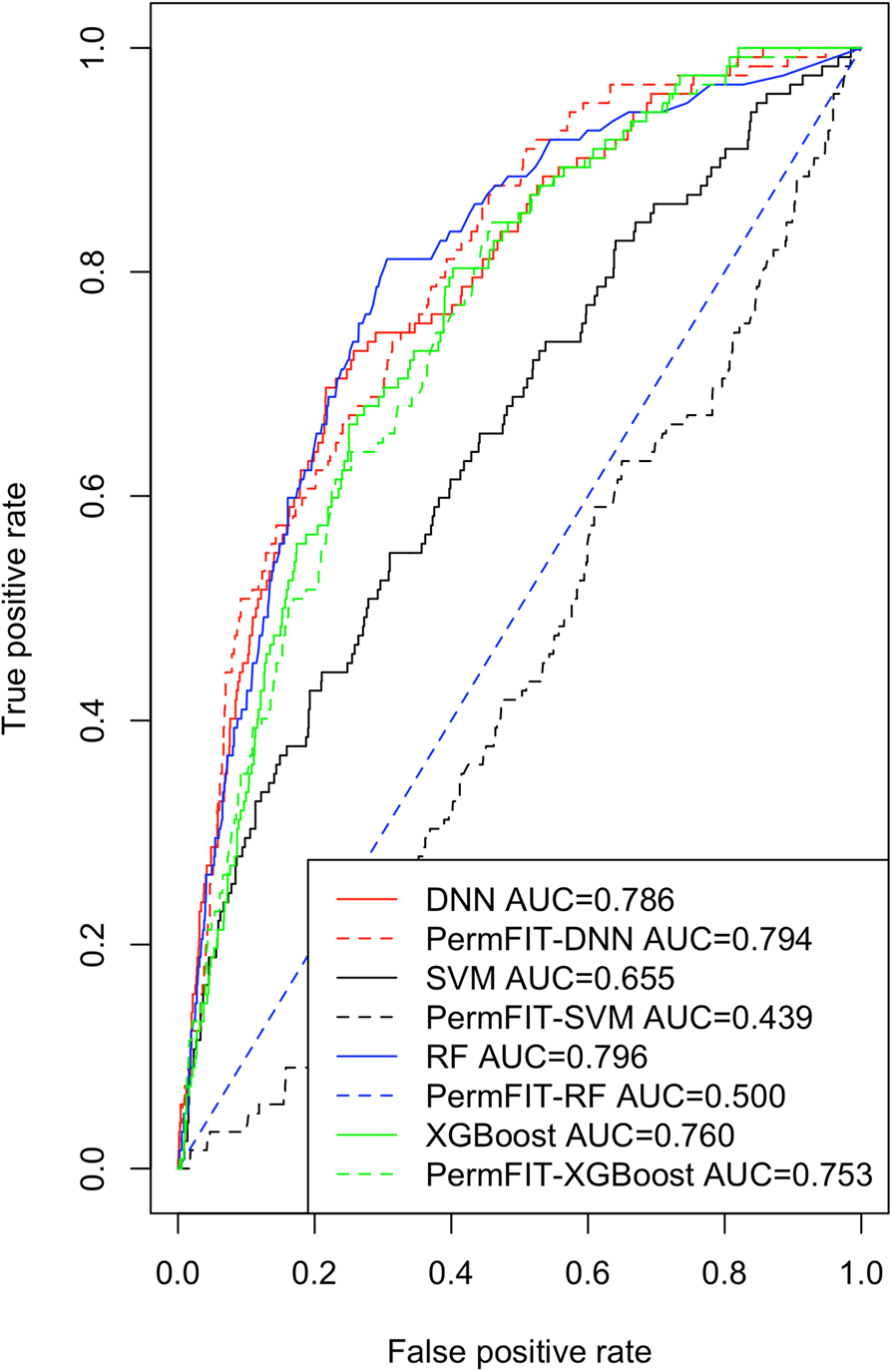
Predicted AUC Comparisons

Results shown in Table 3 demonstrate that all methods have similar performance regarding prediction accuracy. Also, using all 24 features, both the DNN and RF methods have comparable performance on AUC and PR-AUC metrics, but they are all superior to SVM method. However, using the corresponding detected significant features by each method, only the PermFIT-DNN can achieve slightly improved or non-inferior prediction performance for Accuracy, AUC and PR-AUC as compared with the DNN method using all 24 features. We present the predicted AUCs for all methods in Fig. 1. As shown in Table 3, both PermFIT-RF and PermFIT-SVM suffer from remarkably reduced AUC and PR-AUC compared with the corresponding RF and SVM methods using all 24 features. This demonstrates that PermFIT-DNN is far more powerful than PermFIT-RF and PermFIT-SVM methods in identifying valid significant feature associated with TBI status. An interpretation is that there exist some complex functional relationships between clinical features and TBI status, which DNN method can be superior to approximate this complex functional relationship through layer by layer non-linear convolutions as supported by the universal approximation theorem. In particular, the PermFIT-RF only identified one significant feature. Using this significant feature only, the predictive model becomes a random classifier leading to poor prediction performance.

**Table 3:** Performance Comparison for TBI Status Prediction

Method	Accuracy(95% CI)	AUC(95% CI)	PR-AUC(95% CI)	Sensitivity(95% CI)	Specificity(95% CI)
DNN$^{a}$	0.915(0.899,0.931)	0.781(0.703,0.859)	0.972(0.954,0.990)	0.034(0.000,0.171)	1.000(0.994,1.000)
PermFIT-DNN$^{b}$	0.915(0.901,0.869)	0.791(0.713,0.869)	0.973(0.955,0.991)	0.048(0.000,0.129)	1.000(0.994,1.000)
RF$^{a}$	0.911(0.895,0.927)	0.653(0.590,0.716)	0.949(0.933,0.965)	0.041(0.000,0.159)	0.996(0.991,1.000)
PermFIT-RF$^{b}$	0.915(0.909,0.921)	0.411(0.354,0.468)	0.878(0.860,0.896)	0.004(0.000,0.055)	1.000(0.996,1.000)
XGBoost$^{a}$	0.899(0.881,0.917)	0.760(0.684,0.836)	0.970(0.952,0.988)	0.107(0.000,0.260)	0.973(0.967,0.979)
PermFIT-XGB$^{b}$	0.910(0.894,0.926)	0.753(0.679,0.827)	0.968(0.946,0.990)	0.107(0.000,0.225)	0.985(0.975,0.995)
SVM$^{a}$	0.914(0.900,0.928)	0.798(0.718,0.878)	0.973(0.955,0.991)	0.016(0.000,0.151)	0.998(0.994,1.000)
PermFIT-SVM$^{b}$	0.915(0.911,0.919)	0.720(0.655,0.785)	0.957(0.945,0.969)	0.004(0.000,0.022)	1.000(0.998,1.000)

a: a: Include all 24 clinical features in prediction model

b: Include identified significant clinical features only in prediction model

Similarly, the PermFIT-SVM only identified 4 significant features which may not be good enough to distinguish TBI patients. Though the prediction performance using the identified significant features by PermFIT-XGB does not suffer remarkable reduction as compared with that from using all features via XGBoost, it is notably worse than that from PermFIT-DNN. Also, PermFIT-XGB just identified 2 significant features. Additionally, we identified the significant features via the traditional logistic regression method. They include 7 significant features: the children’s age in months, presence of any post-traumatic seizure, duration of any post-traumatic seizure, raised scalp hematoma or swelling, hematoma or swelling location, size of the largest hematoma or swelling, and race. Some of these features overlap with those identified by the PermFIT-DNN method and some do not. However, it should be noted that the traditional parametric method makes a restrictive (generalized) linear additive assumption between the clinical features and TBI status which is unverifiable in practice. This also motivates us to adopt the machine learning methods to relax this restrictive assumption, and thus the identified significant features by the PermFIT-DNN method are more reliable. This observation also indicates that the DNN method not only helps to identify valid significant clinical features via a PermFIT procedure but also using the identified significant features by DNN method, it may improve prediction performance as compared with the DNN model using all clinical features. Very minor or no prediction performance improvement using the identified statistically significant features by the PermFIT-DNN method over using all features via the DNN method implies that the identified significant features provide enough useful information to characterize TBI status, and the other features are nuisance features.

Based on the results of Tables 2 and 3 by using the PermFIT-DNN method, we derive the clinical decision rule of classifying TBI status for suspected minor head trauma of age under 2 year children as: age (in months), acting normally or not based on parental report after head injury, presence or absence of altered mental status according to the ED provider (agitated, sleepy, slow to respond, repetitive questions in the ED), injury mechanism, duration of any post-traumatic seizure, presence of any hematoma(s) or swelling(s) and the location(s) involved, size (diameter) of largest hematoma or swelling, trauma above the clavicles region: scalp-frontal, and trauma above the clavicles region: scalp-parietal, are significant clinical features and should be leveraged and used to determine the TBI status for future children of age under 2 years with suspected minor head injury. ED nurses may focus on examining these significant features to make the triage decision instead of checking all of the available features. It is also worth pointing out that the very minor prediction improvements of PermFIT-DNN over DNN suggest the data set limitation, i.e., uncollected significant features, it is probably the limit of the prediction power it can achieve. However, as shown in the PR-AUC prediction, PermFIT-DNN can achieve over 97% for this highly imbalanced data set indicating the usefulness in clinical practice to identify those true positive TBI infants very accurately.

## Discussion

This study adopted a PermFIT procedure to identify the significant clinical features affecting the TBI status using three commonly used machine learning models with all 24 features as the input. The identified significant features by each machine learning model were used to predict the TBI status via a 10-fold cross-validation and draw comparison with the corresponding machine learning model using all 24 clinical features. The study indicated that the stable DNN method not only outperformed other machine learning models for PR-AUC and comparable accuracy and AUC prediction with RF method, the stable DNN is the most powerful method to identify the significant clinical features. Also, using the identified significant features by the stable DNN can improve the prediction accuracy for AUC and PR-AUC. However, using the identified significant features by RF and SVM models, the predicted AUC and PR-AUC are remarkably reduced as compared to the corresponding predicted values by these two models using all 24 features. All these have clearly demonstrated the benefits of PermFIT-DNN method in clinical practice. Notably, each of the 9 clinical features identified by PermFIT-DNN are consistent with or directly related to clinical features identified as predictors of TBI or clinically important TBI (ciTBI) in prior research and in previously developed clinical decision rules. For example, altered mental status according to the medical provider, child not acting normally according to a parent, an occipital or temporal/parietal hematoma, and severe mechanism of injury (according to PECARN criteria for severity) were all predictors for ciTBI in the PECARN head injury study; [6] altered mental status according to the medical provider, history of seizure following the injury, and presence of swelling over 5 cm were all independent predictors of clinically significant head injury; [15] and altered mental status at 2 hours post-injury, dangerous mechanism of injury (based on similar criteria to PECARN) and large, boggy scalp hematoma were all predictors for the need for neurosurgery or brain injury on CT scan, the primary and secondary outcomes of the CATCH study and resulting clinical decision rule for children under age 16 [14].

Even though the PermFIT-DNN can perform valid statistical inference and offer high prediction accuracy and PR-AUC, the predicted AUC is not very high, which indicates a limitation for this study, i.e., some significant clinical features have not been included in the 24 input features. This suggests that larger scale studies should be conducted to collect more complete clinical features regarding TBI. With more comprehensive clinical features regarding TBI being collected, we expect that more accurate decision rules using the PermFIT-DNN framework can be derived to further improve TBI prediction accuracy. Another limitation of this study is the selection bias that is inherent to any study where only a fraction of the participants receives a diagnostic test, based on the clinician’s assessment of risk for a specific outcome. In the primary study by Kuppermann et al. [6], a CT scan was only obtained if the medical provider deemed it to be warranted. Obtaining CT scans on all children who presented for the evaluation of a head injury would have exposed children to radiation unnecessarily and therefore would have been unethical. The children who did not have a CT scan due to low provider suspicion for ciTBI in the primary study were thus excluded from the current study. Thus, it is plausible that some of the children who did not have a CT scan did in fact sustain a TBI, i.e., some TBI cases were missed.

The results of this study indicate that PermFIT-DNN framework not only robustly identifies valid significant clinical features with solid statistical interpretation but also improves the prediction accuracy with the identified significant clinical features when compared to other machine learning models. The findings of this study could be used to inform the development of clinical decision tools to inform evidence-based clinical decision-making, such as those related to TBI risk and identification. It is worth pointing out that there exist many other machine learning methods that can approximate complex functional relationships accurately. How they perform for identifying significant features under the PermFIT framework deserve further investigation. Also, the PermFIT method can only determine if a feature is significantly associated with the outcome or not under the complex functional relationship. PermFIT can not tell how much the identified significant feature will increase(decrease) the outcome with a unit increase of the feature value. However, this won’t impede the application of PermFIT in clinical practice, e.g., the ED nurses may focus on collecting the 9 identified features for future suspected minor head trauma infants and use them as the input to the trained DNN model to output the accurate probability estimations for these infants to have positive TBIs. Thus, the appropriate triage decisions can be made.

## Data Availability

The data set supporting the conclusions of this article is from the Pediatric Emergency Care Applied Research Network (PeCARN), which is publically available at https://pecarn.org/datasets/. To access this particular data set and other related files, click on the primary manuscript’s title: “Identification of children at very low risk of clinically-important brain injuries after head trauma: a prospective cohort study”.
